# The Effect of Communication Between the Right and Left Liver on the Outcome of Surgical Drainage for Jaundice due to Malignant Obstruction at the Hilus of the Liver

**DOI:** 10.1155/1994/17262

**Published:** 1994

**Authors:** H. U. Baer, M. Rhyner, S. C. Stain, P. W. Glauser, A. R. Dennison, G. J. Maddern, L. H. Blumgart

**Affiliations:** Department of Visceral and Transplantation Surgery University of Berne Inselspital Berne CH-3010 Switzerland

## Abstract

Debate continues regarding the optimal management of irresectable malignant proximal biliary obstruction. Controversy exists concerning the ability of unilateral drainage to provide adequate biliary decompression with tumors that have occluded the communication between the right and left hepatic ductal systems.

Between October 1986 and October 1989, 18 patients with malignant proximal biliary obstruction were treated by an intrahepatic biliary enteric bypass. Patients were divided into two groups based on the presence or absence of a communication between the right and left biliary systems. In Group I (*n* = 9), there was free communication; and in Group II (*n* = 9) there was no communication. There were two perioperative deaths (11%) one due to persistent cholangitis and the other to myocardial insufficiency both with one death in each group. The median survival (excluding perioperative deaths) was 5.6 months. Comparison of pre- and postoperative serum levels of bilirubin and alkaline phosphatase showed a significant decrease in each group, but no difference between the groups in the size of the reduction. Sixteen patients survived at least three months and the palliation was judged as excellent in eight, fair in five, and unchanged in three.

These results demonstrate the effectiveness of biliary enteric bypass regardless of communication between the left and right biliary ductal systems.


*HPB Surgery*, 1994, Vol. 8, pp. 27–31
Reprints available directly from the publisher
Photocopying permitted by license only

© 1994 Harwood Academic Publishers GmbH
Printed in Malaysia

### The Effect of Communication Between the Right and Left Liver on the Outcome of Surgical Drainage for Jaundice due to Malignant Obstruction at the Hilus of the Liver

H. U. BAER, M. RHYNER, S. C. STAIN, P. W. GLAUSER, A. R. DENNISON, G. J. MADDERN and L. H. BLUMGART

Department of Visceral and Transplantation Surgery, University of Berne, Inselspital, CH-3010 Berne, Switzerland

Debate continues regarding the optimal management of irresectable malignant proximal biliary obstruction. Controversy exists concerning the ability of unilateral drainage to provide adequate biliary decompression with tumors that have occluded the communication between the right and left hepatic ductal systems.

Between October 1986 and October 1989, 18 patients with malignant proximal biliary obstruction were treated by an intrahepatic biliary enteric bypass. Patients were divided into two groups based on the presence or absence of a communication between the right and left biliary systems. In Group I ($n = 9$), there was free communication; and in Group II ($n = 9$) there was no communication. There were two perioperative deaths (11%) one due to persistent cholangitis and the other to myocardial insufficiency both with one death in each group. The median survival (excluding perioperative deaths) was 5.6 months. Comparison of pre- and postoperative serum levels of bilirubin and alkaline phosphatase showed a significant decrease in each group, but no difference between the groups in the size of the reduction. Sixteen patients survived at least three months and the palliation was judged as excellent in eight, fair in five, and unchanged in three.

These results demonstrate the effectiveness of biliary enteric bypass regardless of communication between the left and right biliary ductal systems.

KEY WORDS: Obstructive jaundice hilar obstruction surgical drainage unilateral bilateral

### INTRODUCTION

Obstructive jaundice resulting from either benign or malignant biliary structure may be relieved by biliary enteric bypass or transtumoral intubational methods[1–6]. When such obstruction involves the confluence of the hepatic ducts at the liver hilus, the irresectable lesion may completely occlude the confluence, and prevent drainage between the right and left hepatic ductal systems. It has been suggested that adequate relief of jaundice requires decompression of both ductal systems[17–19].

The aim of this study was to assess the outcome for patients with malignant biliary obstruction at the hilus treated by a single biliary enteric anastomosis which drained either one or both ductal systems.

### PATIENTS AND METHODS

Between October 1986 and October 1989, 18 patients with obstructive jaundice had a biliary enteric bypass procedure for proven malignant hilar obstruction [Table 1]. There were 13 males and 5 females with a median age of 56 years (range 42–67). The cause of obstruction were cholangiocarcinoma {8}, gallbladder carcinoma {7}, metastatic pancreatic carcinoma {2} and hepatic metastases from colon carcinoma {1}.

Clinical, biochemical, and radiological assessment was performed in all patients including sonography of the biliary tree, and computed tomography. Delineation of the biliary tract by percutaneous transhepatic cholangiography or endoscopic retrograde cholangiography was employed to provide precise information regarding the anatomy of the biliary obstruction.

All patients had a neoplastic process involving the proximal 1/3 of the extrahepatic bile ducts with 9 patients having total occlusion at the confluence of the right and left hepatic ducts.

The site of hilar occlusion and the presence of a communication between the right and left liver was confirmed at operation. An operative biliary enteric bypass to the segment III duct was the treatment of choice using a Roux-en-Y side to side anastomosis. The segment III bypass was originally described by Soupault and Couinaud[14] and has been modified by several authors[1,2,5,7,15,20,21]. Anastomosis to the segment III duct was possible when preoperative studies demonstrated a dilated left hepatic duct in the absence of left lobar atrophy and without significant parenchymal involvement of the left liver. Consequently, 18 patients had a segment III bypass[22]. Two patients had only a segment V approach[23–25].

The patients were retrospectively divided for assessment into two groups based on cholangiographic evidence of communication between the right and left ductal systems. In Group I ($n = 9$), communication was present; and in Group II ($n = 9$), there was complete obstruction.

The patients were reassessed at three months after operation to evaluate clinical symptoms, and by liver function tests. Assessment of symptomatic outcome was determined by asking whether their current level of activity was excellent, fair or poor. Sixteen patients were available for review (8 in each group). The serum bilirubin and alkaline phosphatase were compared to preoperative levels and between the two groups using the Wilcoxon sign rank test and the student's T test.

### RESULTS

There were two 30 day postoperative deaths (11%), with one death from each group. The first was due to

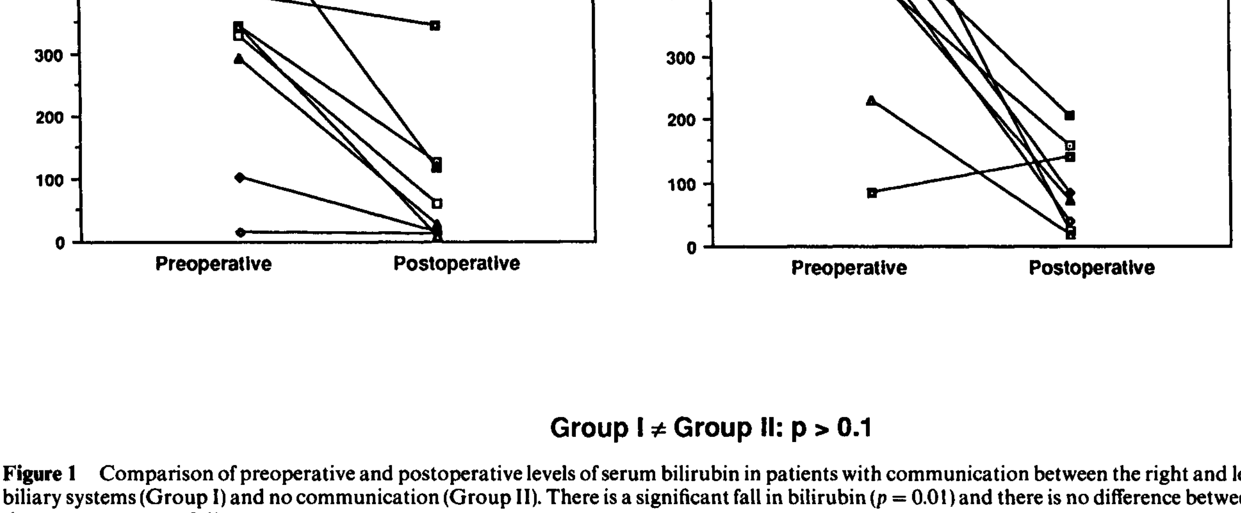

**Figure 1** Comparison of preoperative and postoperative levels of serum bilirubin in patients with communication between the right and left biliary systems (Group I) and no communication (Group II). There is a significant fall in bilirubin ($p = 0.01$) and there is no difference between the two groups ($p = 0.1$).

persistent cholangitis in a patient with a diffuse multicentric cholangiocarcinoma with intrahepatic infiltration. The second patient had gallbladder carcinoma and died of progressive heart failure. Six patients developed complications (33%). Five patients had a pulmonary infiltrate on chest X-ray, and were treated for pneumonia. One patient required reoperation for an early postoperative small bowel obstruction in group I.

The serum bilirubin in Group I before surgery was a median of 303 mmol/l (normal < 25; range 16–545), and at three months after operation, the bilirubin was a median of 101 mmol/l (range 9–334). In Group II, the median serum bilirubin preoperatively was 419 mmol/l (range 84–560), and in three months, had decreased to 110 mmol/l (range 18–253) (Figure 1). In each individual group, the reduction of serum bilirubin was statis tically significant ($p < 0.05$), but there was not a significant difference in the degree of the reduction between the groups.

The fall in the serum level of alkaline phosphatase paralleled that of the bilirubin (Figure 2). There was a significant reduction within each group ($P < 0.05$), and no significant difference in the amount of reduction between Groups I and II. In Group I, the median preoperative alkaline phosphatase value was 783 U/l (range 288–1440; normal < 115), and decreased to 274 U/l (range 46–538). In group II, the median level fell from a median of 534 U/l (range 185–1087) to 316 U/l (range 74–892).

At three months after operation, the quality of palliation was assessed by patient interview. The self described level of activity by the patient was excellent in eight patients, fair in five, and no improvement after operation in three. The median hospital stay for patients discharged from hospital was 25 days (range 12–111 days).

The median survival for the 18 patients discharged from hospital was 5.6 months (range 2–12 months). Examination of the different pathological subsets showed a particularly poor outcome for the three patients with metastatic disease (median survival 3.4 months; range 2–12 months) and the seven patients with gallbladder cancer (median survival 3.8 months: range 2–8 months). The median survival for Group I was 4 months (range 1–14 months), while for Group II it was 9 months (range 0–39 months).

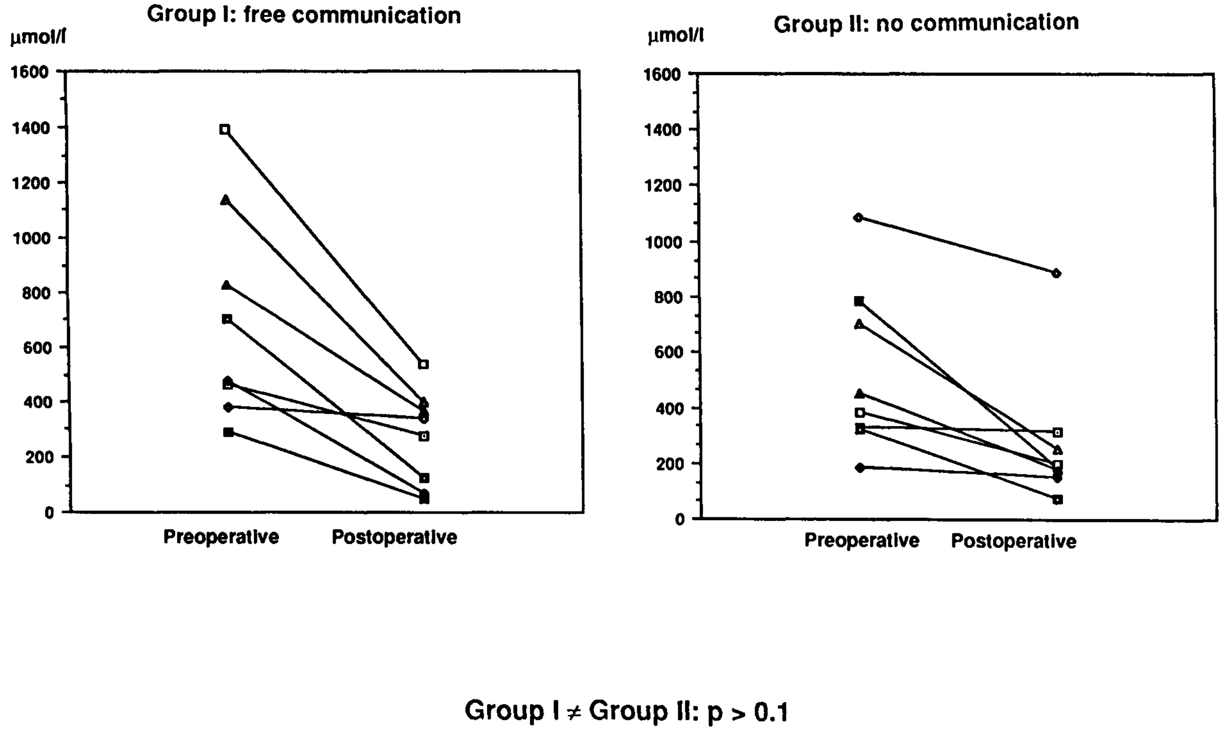

**Figure 2** Comparison of preoperative and postoperative levels of serum alkaline phosphatase in patients with communications between the right and left biliary systems (Group I) and no communication (Group II). There is a significant fall in alkaline phosphatase ($p = 0.01$) and there is no difference between the two groups ($p = 0.1$).

### DISCUSSION

The treatment of malignant proximal biliary obstruction can be difficult due to technical operative problems and the hematological and immunological sequelae of longstanding jaundice[15,26,27,28]. Furthermore, the palliative nature of the treatment, and the availability of transhepatic and endoscopic intubational techniques has resulted in considerable debate regarding the best management of these patients[17,18,19,22,23]. Much of the controversy has centered on the ability of unilateral drainage of the right or left biliary system to provide adequate biliary decompression and subsequent clinical palliation. Indeed, although there has been well documented surgical experience of unilateral drainage, the arguments have persisted, mainly among endoscopists and interventional radiologists, as to whether bilateral drainage is more effective or mandatory. Contributing to the confusion is the lack of documentation in the radiological literature of the presence of lobar liver atrophy in the patients selected for unilateral or bilateral drainage.

The efficacy of palliation by operative biliary enteric bypass has been reconfirmed in the current series. The mortality of 11% is comparable to series of patients treated by operative and nonoperative methods[3,6,15,22]. Thirteen of the 18 patients had self described excellent or fair palliation. Two recent series by Choi *et al.*[6], and Traynor *et al.*[15] examined the results of intrahepatic bypass procedures for patients with hilar obstruction[6–15]. The two studies reported similar results with operative mortality of 6% and 13.6% respectively, and resolution of the jaundice in 73% of patients. The median survival times were 8 and 9 months.

Treatment of irresectable malignant hilar obstruction is palliative. Any efforts to improve the quality of the limited survival should have low morbidity and the necessity of repeated interventions reduced to a minimum. The lessons learned from the results of operative bypass have relevance for patients treated by non operative transtumoral intubational techniques.

Two studies have considered bilateral drainage of the biliary system by endoscopic[29] or the percutaneous route[30] and have failed to show an advantage in either mortality or morbidity when compared with unilateral drainage. These are in contrast to the results of Deviere *et al.*[31] which showed prolonged survival and a low early and late incidence of cholangitis in bilaterally drained patients compared with those in whom only unilateral drainage was possible. They note, however, the poor survival and early cholangitis in undrained segments may have been precipitated by the ERCP itself introducing infection into an undrained or poorly drained segment. Problems with non-operative management of hilar malignancy may be as high as 30%[30] but allows early discharge from hospital. Operative procedures of intrahepatic biliary bypass, while offering lower mortality 6% to 13.6%[6–15] carry a prolonged hospitalization and associated cost, but usually lower the number of readmissions.

Unilateral lobar atrophy when present, may be associated with an ipsilateral dilated hepatic duct[32]. Biliary enteric bypass, or stent placement to the duct draining an atrophied lobe may seem technically inviting, but caution should be exercised since the parenchyma of the atrophied lobe may not have the functional capacity to relieve jaundice[3,22,33]. Furthermore anastomosis to the duct of a nonfunctional atrophied lobe may result in an increased incidence of early and late infectious complications, and limits the effectiveness of palliation[6].

Comparison between the two groups of patients with drainage of a single versus both sides of the liver is illustrative of what can be expected with transtumoral stent placement. The reductions in serum bilirubin and alkaline phosphatase were similar, and there was no difference in mean survival, or in the quality of palliation if hilar communication is present or absent. The demonstration that unilateral drainage produces similar results to bilateral drainage suggests that an increased number of such patients can be offered the option of palliative operative bypass. Complete hilar obstruction is not a contraindication to unilateral operative biliary decompression.

### ACKNOWLEDGEMENTS

We thank Mrs. B. Moser-Etter for her valuable secretarial assistance.

### REFERENCES

1. Blumgart, L. H. (1988) Liver resection—liver and biliary tumours. In Surgery of the liver and biliary tract, edited by L. H. Blumgart, vol. II, pp. 1251–1280. Edinburgh, London, Melbourne, New York: Churchill Livingstone
2. Aranha, G. V., Prin, R. A. and Greenlee, H. B. (1987) Biliary enteric bypass for benign and malignant disease. *Am. Surg.*, **53**, 403–406.
3. Bismuth, H. and Corlette, B. (1975) Intrahepatic cholangioenteric anastomosis in carcinoma of the hilus of the liver. *Surg. Gynecol. Obstet.*, **140**, 170–178.
4. Blumgart, L. H. Benjamin, I. S., Hadjis, N. S. and Beazley, R. (1984) Surgical approaches to cholangiocarcinoma at confluence of hepatic ducts. *Lancet*, **i**, 66–70.

5. Blumgart, L. H. and Kelley, C. (1984) Hepaticojejunostomy in benign and malignant high bile duct structure: Approaches to the left hepatic ducts. *Br. J. Surg.*, **71**, 257–261.
6. Choi, T. K., Fan, S. T., Lai, E. C. S. and Wong, J. (1988) Malignant hilar biliary obstruction treated by segmental bilioenteric anastomosis. *Surgery*, **104**, 525–529.
7. Hepp, J. and Couinaud, C. (1956) L'abord et l'utilisation du canal hépatique gauche dans les réparations de la voie biliaire principale. *Presse Med.*, **64**, 947–948
8. Longmire, W. P. and Lippmann, H. N. (1948) Intrahepatic cholangio-jejunostomy with partial hepatectomy for biliary obstruction. *Surgery*, **24**, 264–276.
9. Longmire, W. P. and Lippmann, H. N. (1956) Intrahepatic cholangiojejunostomy—an operation for biliary obstruction. *Surg. Clin. North Am.*, **36**, 849–863.
10. Malt, R. A., Warshaw, A. L., Jamieson, C. G. and Hawk, J. C. (1980) Left intrahepatic cholangiojejunostomy for proximal obstruction of the biliary tract. *Surg. Gynecol. Obstet.*, **150**, 193–197.
11. Mizumoto, R., Kawarada, Y. and Suzuki, H. (1986) Surgical treatment of hilar carcinoma of the bile duct. *Surg. Gynecol. Obstet.*, **162**, 153–158.
12. Moreno-Gonzales, E., Sanmartin, J. H., Solis Herruzo, J. A. and Moreno Ascoita, E. (1980) Intrahepatic biliary intestinal diversion for biliary obstruction: experience in 34 patients. *Br. J. Surg.*, **67**, 263–265.
13. Ragis, H., Diamond, A. and Meng, C. (1973) Intraoperative cholangio-jejunostomy in the management of malignant biliary obstruction. *Surg. Gynecol. Obstet.*, **136**, 27–32.
14. Soupault, R. and Couinaud, C. (1957) Sur un procède nouveau de dérivation biliare intrahépatique: la cholangio-jejunostomy gauche sans sacrifice hépatique. *Presse Med.*, **65**, 1157–1159.
15. Traynor, O., Castaing, D. and Bismuth, H. (1987) Left intrahepatic cholangioenteric anastomosis (round ligament approach) an effective palliative treatment for hilar cancers. *Br. J. Surg.*, **74**, 952–954.
16. Hjortsjoe, C. H. (1951) The topography of the intrahepatic duct system. *Acta Anatomica*, **11**, 599–615.
17. Soehendra, N., Grimm, H., Berger, B. and Nam, V. C. (1989) Malignant jaundice: results of diagnostic and therapeutic endoscopy. *World J. Surg.*, **13**, 171–177.
18. Grimm, H. and Soehendra, N. (1984) Endoscopic biliary drainage (Hamburg). In: Hepato-biliary and pancreatic malignancies, edited by N. J. Lygidakis, G. N. J. Tytgat, pp. 418–425. Stuttgart, New York: G. Thieme Verlag.
19. Huibregtse, K. and Tytgat, G. N. Endoscopic biliary drainage (Amsterdam). In: Hepatobiliary and pancreatic malignancies, edited by N. J. Lygidakis, G. N. J. Tytgat, pp. 426–438 Stuttgart, New York: G. Thieme Verlag.
20. Couinaud, C. (1981) Exposure of the intrahepatic bile duct, controlled hepatectomies and exposure of the intrahepatic bile ducts. Anatomical and technical study. Paris.
21. Couinaud, C. (1989) Exposure of the left hepatic duct through the hilum or in the umbilical of the liver: Anatomic limitations. *Surgery*, **105**, 21–27.
22. Blumgart, L. H. (1988) Hilar and intrahepatic biliary-enteric anastomosis. In Surgery of the liver and biliary tract, edited by L. H. Blumgart, vol. II, pp. 911–913. Edinburgh, London, Melbourne, New York: Churchill Livingstone.
23. Blumgart, L. H. (1988) Right-sided intrahepatic hepaticojejunostomy. In Surgery of the liver and biliary tract, edited by L. H. Blumgart,vol. II, pp. 911–913. Edinburgh, London, Melbourne, New York: Churchill Livingstone.
24. Blumgart, L. H. and Thompson, J. N. (1987) Right-sided intra hepatic hepaticojejunostomy. *Curr. Probl. Surg.*, **24**, 42–43.
25. Hess, W. (1986) Intrahepatische biliodigestive Anastomosen (Cholangioenterostomien). In Die Erkrankungen der Gallenwege und des Pankreas, edited by W. Hess *et al.* Band 2, pp. 1581–1584. Padova: Piccin Nuova Libreria.
26. Halliday, A. W., Benjamin, I. S. and Blumgart, L. H. (1988) Nutritional risk factors in major hepatobiliary surgery. *J. Parent. Ent. Nutr.*, **12**, 43–48.
27. Pitt, H. A., Cameron, J. L., Postie, R. G. and Gadacz, T. R. (1981) Factors affecting mortality in biliary tract surgery. *Am. J. Surg.*, **141**, 66–72.
28. Thompson, J. N., Cohen, J., Moore, R. H., Blenkharn, J. I., McConnell, J. S., Matkin, J. and Blumgart, L. H. (1988) Endo toxemia in obstructive jaundice. *Am. J. Surg.*, **155**, 314–321.
29. Polydorou, A. A., Chisholm, E. M., Romanos, A. A., Dowsett, J. F., Cotton, P. B., Hatfield, A. R. W. and Russel, R. C. G. (1989) A comparison of right versus left hepatic duct endoprosthesis insertion in malignant hilar biliary obstruction. *Endoscopy*, **21**, 266–271.
30. Hall, R. I., Denyer, M. E. and Chapman, A. H. (1990) Percutaneous-endoscopic placement of endoprostheses for relief of jaundice caused by inoperable bile duct strictures. *Surgery*, **107**, 224–227.
31. Deviere, J., Baize, M., de Toeuf, J. and Cremer, M. (1988) Longterm follow-up of patients with hilar malignant strcture treated by endoscopic internal biliary drainage. *Gastrointest. Endosc.*, **34**, 95–101.
32. Hadjis, N. S. and Blumgart, L. H. Liver hyperplasia, hypertrophy and atrophy; clinical relevance. In Surgery of the liver and biliary tract, edited by L. H. Blumgart, vol. I, pp. 61–71. Edinburgh, London, Melbourne, New York: Churchill Livingstone.
33. Bismuth, H. and Malt, R. A. (1979) Carcinoma of the biliary tract. *New. Engl. J. Med.*, **301**, 704–706.

